# Uncommon P1 Anchor-featured Viral T Cell Epitope Preference within HLA-A*2601 and HLA-A*0101 Individuals

**DOI:** 10.4049/immunohorizons.2400026

**Published:** 2024-06-17

**Authors:** Jianing Zhang, Can Yue, Yin Lin, Jinmin Tian, Yuanyuan Guo, Danni Zhang, Yaxin Guo, Beiwei Ye, Yan Chai, Jianxun Qi, Yingze Zhao, George F. Gao, Zeyu Sun, Jun Liu

**Affiliations:** * National Key Laboratory of Intelligent Tracking and Forecasting for Infectious Diseases (NITFID), National Institute for Viral Disease Control and Prevention, Chinese Center for Disease Control and Prevention, Beijing, China; † NHC Key Laboratory of Biosafety, Research Unit of Adaptive Evolution and Control of Emerging Viruses, Chinese Academy of Medical Sciences, National Institute for Viral Disease Control and Prevention, Chinese Center for Disease Control and Prevention, Beijing, China; ‡ CAS Key Laboratory of Infection and Immunity, National Laboratory of Macromolecules, Institute of Biophysics, Chinese Academy of Sciences (CAS), Beijing, China; § Department of Epidemiology, School of Public Health, Cheeloo College of Medicine, Shandong University, Jinan, Shandong Province, China; ¶ CAS Key Laboratory of Pathogen Microbiology and Immunology, Institute of Microbiology, Chinese Academy of Sciences (CAS), Beijing, China; ‖ State Key Laboratory for Diagnosis and Treatment of Infectious Diseases, National Clinical Research Center for Infectious Diseases, The First Affiliated Hospital, School of Medicine, Zhejiang University, Hangzhou, China

## Abstract

The individual HLA-related susceptibility to emerging viral diseases such as COVID-19 underscores the importance of understanding how HLA polymorphism influences peptide presentation and T cell recognition. Similar to HLA-A*0101, which is one of the earliest identified HLA alleles among the human population, HLA-A*2601 possesses a similar characteristic for the binding peptide and acts as a prevalent allomorph in HLA-I. In this study, we found that, compared with HLA-A*0101, HLA-A*2601 individuals exhibit distinctive features for the T cell responses to SARS-CoV-2 and influenza virus after infection and/or vaccination. The heterogeneous T cell responses can be attributed to the distinct preference of HLA-A*2601 and HLA-A*0101 to T cell epitope motifs with negative-charged residues at the P1 and P3 positions, respectively. Furthermore, we determined the crystal structures of the HLA-A*2601 complexed to four peptides derived from SARS-CoV-2 and human papillomavirus, with one structure of HLA-A*0101 for comparison. The shallow pocket C of HLA-A*2601 results in the promiscuous presentation of peptides with “switchable” bulged conformations because of the secondary anchor in the median portion. Notably, the hydrogen bond network formed between the negative-charged P1 anchors and the HLA-A*2601-specific residues lead to a “closed” conformation and solid placement for the P1 secondary anchor accommodation in pocket A. This insight sheds light on the intricate relationship between HLA I allelic allomorphs, peptide binding, and the immune response and provides valuable implications for understanding disease susceptibility and potential vaccine design.

## Introduction

MHC, also known as HLA in humans, plays a crucial role in orchestrating the immune system recognition and response to viral pathogens (1). For instance, HLA-B*1501 is associated with asymptomatic SARS-CoV-2 infection due to preexisting T cell immunity to OC43-CoV and HKU1-CoV (2). In addition to cellular immunity, it was also demonstrated that the Ab levels were positively associated with the presence of the HLA-A*0301 allomorph with BNT162b2 vaccine (3). However, viruses can also downregulate MHC class I (MHC I) expression to evade elimination, which indicates the pivotal role of MHC I in antiviral immunity. SARS-CoV-2 ORF7a interacts with MHC I H chains in the endoplasmic reticulum and slows the export of MHC I/peptide complexes (4). The ongoing warfare between viruses and human immunity is never-ending (5).

An analysis of more than 8 million population from 20 registries worldwide, conducted by the 18th International HLA & Immunogenetics Workshop, revealed that HLA-A*0101 accounted for 13.34%, ranking second only to HLA-A*0201 (24.06%), whereas HLA-A*2601 (3.35%) was the seventh most common allele globally (6). Due to the large HLA polymorphism, Sette et al. defined the HLA allelic variants that bind peptides possessing a particular HLA supermotif as being collectively referred to as an HLA superfamily (7), with nine major HLA class I supertypes of HLA-A and -B (i.e., A1, A2, A3, A24, B7, B27, B44, B58, and B62). On the basis of common alleles and well-documented alleles in China, the A3 superfamily (44.07%), A2 superfamily (30.12%), and A24 superfamily (16.44%) represent the top three common HLA allele superfamilies, followed closed by the A1 superfamily (8.57%). HLA-A*0101 (3.40%) is the most frequent allele in the HLA-A1 superfamily in China (8, 9), especially in the northeast of China. In Jilin Province, the frequency of HLA-A*0101 is 4.63% (10), and the frequency of HLA-A*0101 is 4.89% in Heilongjiang Province (11).

HLA-A*0101 and HLA-A*2601 have been associated with a variety of diseases. For example, HLA-A*2601 is considered a susceptible allele in HLA-B*51X noncarrier patients with Behçet’s disease (12) and may be considered as a protective allele against pemphigus vulgaris in Iranian patients (13). It is also significantly increased in idiopathic hypoparathyroidism compared with controls (14). On the other hand, HLA-A*0101 may be linked to psoriatic arthritis (15, 16) and serves as the susceptibility gene for patients with cervical cancer in Xinjiang, China (17). It is also associated with Löfgren’s syndrome in the Czech population (18) and is a genetic marker for an increased risk of Graves’ orbitopathy (19). HLA-A*0101 has been implicated in phenobarbital hypersensitivity in Thai children (20) and is associated with an increased risk of EBV positivity (21). Furthermore, HLA-A*0101 is linked to high-risk clonal evolution in immunosuppressive therapy for acquired aplastic anemia (22). However, it appears to be a less specific molecular mechanism of HLA as a risk or protective factor for these diseases.

A previous study targeted HLA-A26^+^ patients with cancer with a peptide vaccine, resulting in a 100% enhancement of peptide-specific IgG levels in all participants after two vaccination cycles (23). Kongkaew et al. conducted molecular docking simulations of peptides related to Behcet’s disease with HLA-A*2601 (24). Prior studies have shown that pocket B of the HLA-A*0101 prefers small and aliphatic amino acids, whereas pocket F favors aromatic and large hydrophobic amino acids (8). However, it remains unclear how the peptide binding and presentation of HLA-A*0101 and HLA-A*2601 influence their different disease sensitivity and immune responses.

In this study, we uncovered the different T cell epitope recognition feature of HLA-A*2601, compared with HLA-A*0101, through a series of functional and structural investigations. Our findings indicate the structural basis and T cell response phenotype of HLA-A*2601 to dominantly prefer peptides with acidic amino acids D and E preference at the P1 position. Our results increase the understanding of HLA-A superfamilies and provide a method for peptide screening and vaccine development based on the binding motifs of different allomorphs.

## Materials and Methods

### PBMCs from donors

PBMC samples from donors who have recovered from COVID-19 were collected. Standard serologic HLA typing was performed with peripheral blood from the donors by BGI Tech Solutions (Beijing Liuhe) Co. Donors were categorized into three groups: an HLA-A*2601^+^ group, an HLA-A*0101^+^ group, and an HLA-A*2601^+^HLA-A*0101^+^ group. Detailed information is provided in Table I. The study was approved by the ethics committee of the National Institution for Viral Disease Control and Prevention, China CDC. All of the subjects signed an informed consent before the study.

**Table I.** Characteristics of the subjects used in this study

**Donor group**	**ID**	**Sex**		**HLA allele**						**COVID-19**		**Influenza**			**HAI titer**			**Time of sample collection**		
				**HLA-A**			**HLA-B**		**Infection** **(if infection, time*^a^*)**		**Vaccine dose** **(time of last dose)**	**Infection in last three months**		**Vaccine dose** **(time of last dose)**		**H1N1*^b^***	**H3N2*^c^***			
HLA-A*2601^+^	1	Female	A*2601		A*2402	B*1518		B*3503	Yes (1/6/2023)		4 (5/2022)	Not clear	4 (4/2022)			20	20		6/15/2023
	2	Female	A*2601		A*1101	B*1558		B*3801	Yes (12/2022)		4 (5/2023)	Not clear	0			10	40		
	3	Female	A*2601		A*1101	B*1501		B*4001	Yes (12/2022)		2 (6/2021)	Yes	1 (11/2022)			640	640		
	4	Male	A*2601		A*0201	B*1518		B*4003	Yes (12/2022)		3 (10/2022)	Yes	3 (10/2021)			10	10		
	5	Male	A*2601		A*1101	B*4006		B*5001	Yes (12/2022)		2 (2021)	Not clear	Not clear			20	40		
	36	Female	A*2601		A*1101	B*1527		B*4006	Yes (12/2022)		3 (12/2022)	No	1 (12/2022)			640	1280		
HLA-A*0101^+^	6	Male	A*0101		A*0203	B*3701		B*3802	No		4 (12/2022)	No	0			80	80		
	7	Female	A*0101		A*1101	B*1517		B*5401	Yes (12/2022)		2 (2/2022)	No	0			5	10		
	8	Male	A*0101		A*2901	B*0705		B*1501	Yes (5/2023)		4 (5/2023)	Not clear	Not clear			640	640		
	9	Male	A*0101		A*2402	B*0702		B*6701	Yes (12/2022)		3 (12/2021)	No	0			5	10		
	37	Female	A*0101		A*0207	B*3701		B*4601	Yes (5/2023)		0	No	0			40	40		
	38	Female	A*0101		A*0201	B*4403		B*6701	No		4 (1/2023)	No	1 (11/2022)			160	320		
HLA-A*2601^+^ HLA-A*0101^+^	34	Male	A*0101		A*2601	B*3701		B*4001	Yes (5/2023)		4(5/2023)	No	1 (10/2022)			1280	40		

HAI, HA inhibition.

a Donors were all were mild healthy individuals, and time refers to the self-reported time of the latest symptom onset before sample collection.

b Recommended composition of influenza virus vaccines for use in the 2022–2023 Northern Hemisphere influenza season: A/Victoria/2570/2019 (H1N1)pdm09-like virus EPI_ISL_417210. Cutoff value 20.

c Recommended composition of influenza virus vaccines for use in the 2021–2022 Northern Hemisphere influenza season: A/Cambodia/e0826360/2020 A(H3N2) EPI_ISL_80654. Cutoff value 20.

### In vitro stimulation and culture of PBMCs

The cells were thawed and incubated with 10 μM peptides in RPMI 1640 containing 10% FBS at 37°C with 5% CO_2_ at a cell density of 2 × 10^6^/ml in a 48-well culture plate, and 25 ng/ml recombinant human IL-7 was added to the medium. A peptide pool with a concentration of 2 μg/ml for each peptide was used for the Ag-specific T cell proliferation. On day 3 and day 6, half of the medium was changed with 20 U/ml recombinant human IL-2, and the supplement with an equal amount of peptide was performed. On day 9, cells were harvested and tested for the presence of peptide-specific T cells by ELISPOT assay.

### ELISPOT assay

The Ag-specific response of T lymphocytes induced by peptides was measured by the use of an IFN-γ ELISPOT set (BD Biosciences). Briefly, a 96-well ELISPOT plate membrane was preincubated with diluted anti-IFN-γ coating Ab overnight at 4°C. The next day, wells were washed with RPMI 1640 and blocked for 3 h at 37°C. PBMCs from donors were incubated in microwells (1 × 10^5^/well) along with stimulating peptides (20 μM) or PMA as a positive control of nonspecific stimulation for 18 to 24 h at 37°C with 5% CO_2_. Cells incubated without a stimulator were employed as a negative control. Subsequently, the cells were removed, and the plate was processed in turn with biotinylated detection Abs, streptavidin-HRP conjugate, and substrate 3-amino-9-ethylcarbazole. Development of colored spots was stopped by thorough rinsing with demineralized water. The results were analyzed using an automatic ELISPOT reader (CTL2.7).

### Peptide synthesis and expression constructs of proteins

To screen peptides for binding to HLA-A*2601 and HLA-A*0101, SARS-CoV-2 (GenBank no. NC_045512.2), influenza A virus (IAV) (PB1: GenBank no. NC_026435.1, NP: GenBank no. NC_026436.1), and human papillomavirus (HPV) type 16 (L1: GenBank no. ACG75893.1) were used to screen peptides. The conserved and functional T cell epitope peptides were selected according to the Immune Epitope Database and Tools (https://www.iedb.org/). Peptides were synthesized and purified through analytical HPLC and mass spectrometry (Scilight Biotechnology, LLC), with the purity as ∼95%. The peptides were stored at −80°C as freeze-dried powders and were dissolved in DMSO before use. Detailed information of the peptides is shown in Table II.

**Table II.** Characteristics of the peptides used in this study

**Virus**	**Peptide name**	**Protein**	**Sequence**	HLA-A*0101 positive	HLA-A*2601 positive	**References**
SARS-CoV-2	STS	ORF1ab 483–491	STSAFVETV	**+**	**+**	(54)
	YTT	ORF1ab 1873–1882	YTTTIKPVTY	**+**	**+**	(48, 49)
	EID	ORF1ab 1891–1899	EIDPKLDNY	**+**	**+**	(48)
	CTE-11	ORF1ab 1889–1899	CTEIDPKLDNY	**+**		(49)
	EVG	ORF1ab 2089–2099	EVGHTDLMAAY		**+**	(48)
	NST-10	ORF1ab 2272–2281	NSTNVTIATY		**+**	(48)
	STN	ORF1ab 2273–2281	STNVTIATY	**+**		(48)
	SEF	ORF1ab 3946–3954	SEFSSLPSY	**+**	**+**	(54)
	VAT	M 170–178	VATSRTLSY	**+**	**+**	(54)
	YTN-10	S 28–37	YTNSFTRGVY	**+**	**+**	(48)
	YTN-11	S 28–38	YTNSFTRGVYY	**+**		(49)
	NSF	S 30–38	NSFTRGVYY		**+**	(48)
	WTA	S 258–266	WTAGAAAYY	**+**	**+**	(48, 55)
	EVF	S 340–351	EVFNATRFASVY		**+**	(48)
	NST	S 343–351	NSTRFASVY	**+**		(56)
	CVA	S 361–369	CVADYSVLY	**+**	**+**	(48, 55)
	TSN	S 604–612	TSNQVAVLY	**+**	**+**	(48, 56)
IAV	HSN	NP 140–148	HSNLNDATY	**+**	**+**	(57)
	YSH	PB1 30–38	YSHGTGTGY	**+**	**+**	(57)
	LVS	PB1 590–599	LVSDGGPNLY	**+**	**+**	(57)
	CTE	NP 44–52	CTELKLSDY	**+**		(56)
HPV-16	DVM	L1 412–420	DVMTYIHSM		**+**	Screening from our laboratory
	YVA	L1 53–61	YVARTNIYY		**+**	

The expression plasmids for the extracellular domains of HLA-A*2601 (GenBank no. AAA03720.1) and HLA-A*0101 (GenBank no. CAB93537.1) were synthesized (Nanjing Genscript Biotechnology Co.) and constructed in PET-28a (+) vector. The expression plasmid for human β_2_-microglobulin (β_2_m) was constructed previously in our laboratory (25).

### Refolding and purification of HLA-A*2601 complexes

The soluble monomer proteins of HLA-A*2601 and HLA-A*0101 complexed to β_2_m and their specific peptides were produced as described previously (26). Briefly, HLA-A*2601 H chain and β_2_m were overexpressed as recombinant proteins in *Escherichia coli* BL21(DE3) and subsequently in vitro refolded and assembled in the presence of a high concentration of (or without) peptides. Generally, the refolding buffer was 200 ml, and the molar ratio of peptide, β_2_m, and H chain was 1:1:3. After sufficient time for protein refolding, the buffer was concentrated and analyzed by Superdex 200 10/300 GL gel filtration chromatography (GE Healthcare).

### Determination of protein thermostability using circular dichroism spectroscopy

The thermostabilities of HLA-A*0101 and HLA-A*2601 complexed with peptides were tested by circular dichroism (CD) spectroscopy. All complexes were refolded, purified, and measured at 0.2 mg/ml. CD spectra at 218 nm were measured on a Chirascan spectrometers (Applied Photophysics) using a thermostatically controlled cuvette at temperature intervals of 0.2°C at an ascending rate of 1°C/min between 20°C and 90°C. The unfolded fraction (percentage) is expressed as (θ − θ_a_)/(θ_a_ − θ_b_), where θ_a_ and θ_b_ are the mean residue ellipticity values in the fully folded and fully unfolded states. The denaturation curves were generated by nonlinear fitting with OriginPro. The *T_m_* was calculated by fitting data to the denaturation curves and using inflection-determining derivatives.

### Crystallization, data collection, and processing

The proteins were diluted with pool buffer at a 1:1 ratio and crystallized by using the sitting-drop gas-phase diffusion technique at 18°C. A crystal screen kit (Hampton Research) was used to screen for optimal crystal growth conditions of the HLA-A*2601 complexes. HLA-A*2601/DVM crystals were grown in PEGRxI. HLA-A*2601/YVA crystals were grown in PEGRx 1. HLA-A*2601/EVF crystals were grown in 0.1 M Bis-Tris, pH 6.5, 20% polyethylene glycol monomethyl ether 5000 at a protein concentration of 14.3 mg/ml. HLA-A*2601/CVA crystals were grown in 0.1 M imidazole, pH 7.0, 20% w/v polyethylene glycol 6000 at a protein concentration of 16.7 mg/ml. HLA-A*0101/EVF-F3D crystals were grown in 0.2 M potassium chloride, 20% w/v polyethylene glycol 3350 at a protein concentration of 6.8 mg/ml. Crystals were placed in a solution containing 20% glycerol as antifreeze and then flash-cooled in a 100-K gaseous nitrogen stream. The X-ray diffraction data were collected in BL02U1 and BL19U1 beamline stations at Shanghai Synchrotron Radiation Facility (Shanghai, China).

The collected intensities were subsequently processed and scaled using the HKL2000 software package. The structures were determined using molecular replacement with the program Phaser MR in Phenix (27). The model used was the structure coordinates with Protein Data Bank (PDB) code 4NQV (28). Extensive model building was performed by hand using Coot (29). The stereochemical quality of the final model was assessed with the program REFINE in Phenix (30). Structure-related figures were generated using PyMOL (PyMOL Molecular Graphics System, version 2.5.0a0, Open-Source Schrödinger, LLC).

### Elution, mass spectrometry, and analysis of random peptide library

The HLA-A*2601/peptide and HLA-A*0101/peptide complexes refolded by a random 11-mer peptide were condensed to 7 mg/ml, and 2 M acetic acid was added and incubated at 90°C for 5 min. The solution was filtered with a centrifugal filter unit with a molecular mass cutoff size of 3 kDa. The obtained peptides were obtained by liquid chromatography–tandem mass spectrometry. The peptide sequence was determined by de novo sequencing and was used to generate a sequence logo diagram using Weblogo2.

### Accession numbers

Atomic coordinates and structure factors have been deposited in the PDB under accession codes 8XES for HLA-A*2601/DVM, 8XFZ for HLA-A*2601/YVA, 8XG2 for HLA-A*2601/EVF, 8XKC for HLA-A*2601/CVA, and 8XKE for HLA-A*0101/EVF-F3D.

## Results

### Preference of binding peptide motifs of HLA-A*2601 and HLA-A*0101

**FIGURE 1.**
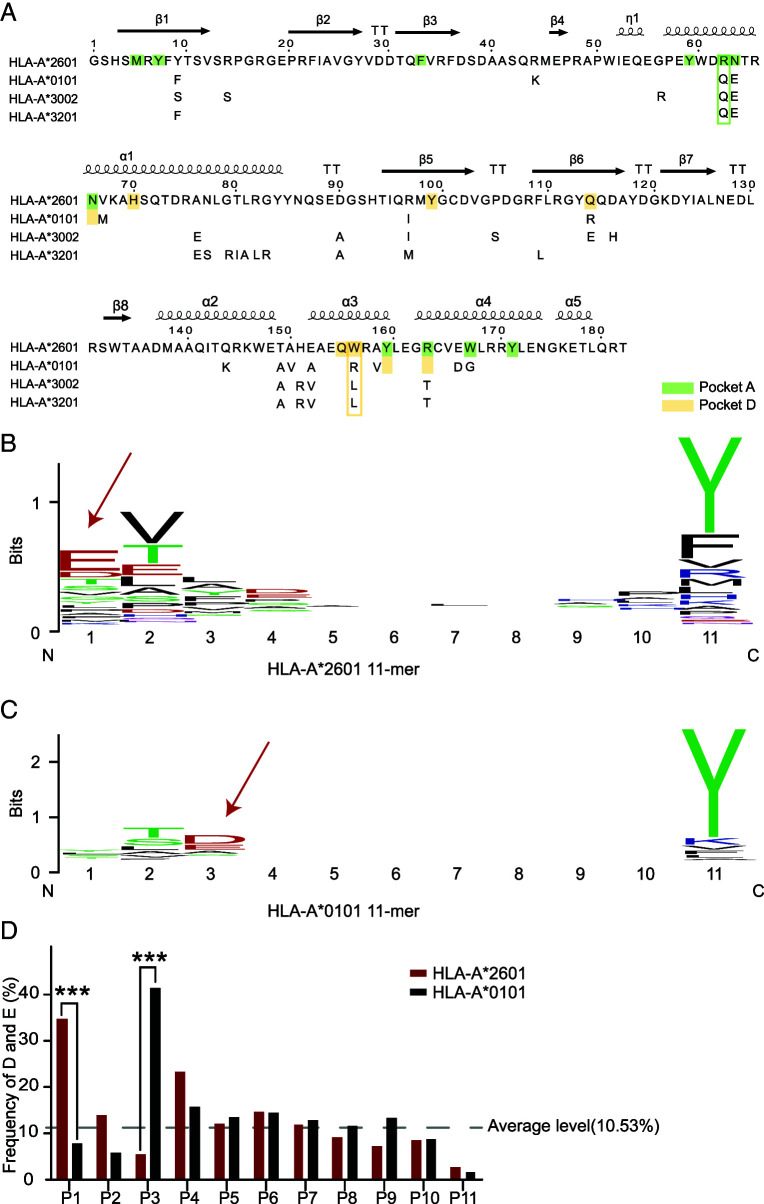
Sequence and random peptides characteristics of HLA-A*2601 and HLA-A*0101. (**A**) **α**1 and **α**2 domain sequence alignment among HLA-I allomorphs. MEGA (51) and ESPript (52) were used to generate sequence alignments. Key amino acids affecting pocket recognition residues are shown in the box. HLA-A*2601 GenBank no. AAA03720.1; HLA-A*0101 GenBank no. CAB93537.1; HLA-A*3002 GenBank no. AAF73068.1; HLA-A*3201 GenBank no. CAD87772.1. (**B**) Random 11-mer peptide results for HLA-A*2601. (**C**) Random 11-mer peptide results for HLA-A*0101. The sequence conservation at each position is indicated by the total height of the stack, and the relative frequency of each amino acid at this position is indicated by the height of the symbol within the stack. Blue letters, positive amino acids; red letters, negative amino acids. Weblogo was used to generate sequence logo (53). (**D**) The frequency of D and E in random peptide library in each position of peptides. The χ^2^ test was performed using GraphPad Prism version 9.5.1. ***p <* *0.001.

Between HLA-A*2601 and HLA-A*0101, there are several amino acids with polymorphisms. Key residues Tyr9, Arg62, Asn63, Arg97, Gln114, Thr149, Glu152, and Trp156 are unique to HLA-A*2601 in the α1 and α2 domains. Among them, two of the amino acids, Arg62 and Asn63, make up pocket A, and Trp156 contributes to the forming of pocket D (Fig. 1A) as the conformation of traditional HLA-I.

To evaluate the peptide presentation influenced by the minor amino acid polymorphism within the peptide binding grooves (PBGs), a random 11-mer peptide library (without Cys) was synthesized. The library was used to assist the renaturation of HLA-A*2601 and HLA-A*0101 with β_2_m. The peptides that bound to HLA were eluted with glacial acetic acid and were analyzed by de novo sequencing analysis via liquid chromatography–tandem mass spectrometry. The random peptide results show that HLA-A*2601 and HLA-A*0101 share similar binding peptide motifs, with P2 and P_Ω_ (C-terminal position) positions serving as the major anchor residues. The P2 position prefers Val or Thr, whereas the P_Ω_ position prefers an aromatic hydrophobic amino acid such as Tyr. However, distinguishing features can be observed in the P1 and P3 positions of the peptide motifs of the two molecules. The P1 of HLA-A*2601 presented peptides prefer the negative-charged residues, whereas the P3 of HLA-A*0101-binding peptides prefer negative charges (Fig. 1B, 1C). Generally, if one position contains a random residue of the 19 candidate amino acids (without Cys), the average frequency would be 5.26% for each amino acid, and the probabilities of D and E appearing are 10.53%. For P1 of HLA-A*2601-binding peptides, the frequency of D and E occurring is 34.66%, whereas for P3 of HLA-A*0101 peptides, it is 41.21% (Fig. 1D). Thus, it can be inferred that HLA-A*2601 and HLA-A*0101 exhibit distinctive binding motifs at the P1 and P3 positions by favoring negative charges, respectively, which may play an important role in HLA recognition.

### Different T cell responses to epitopes of SARS-CoV-2 and influenza A virus among HLA-A*2601 and HLA-A*0101 donors

**FIGURE 2.**
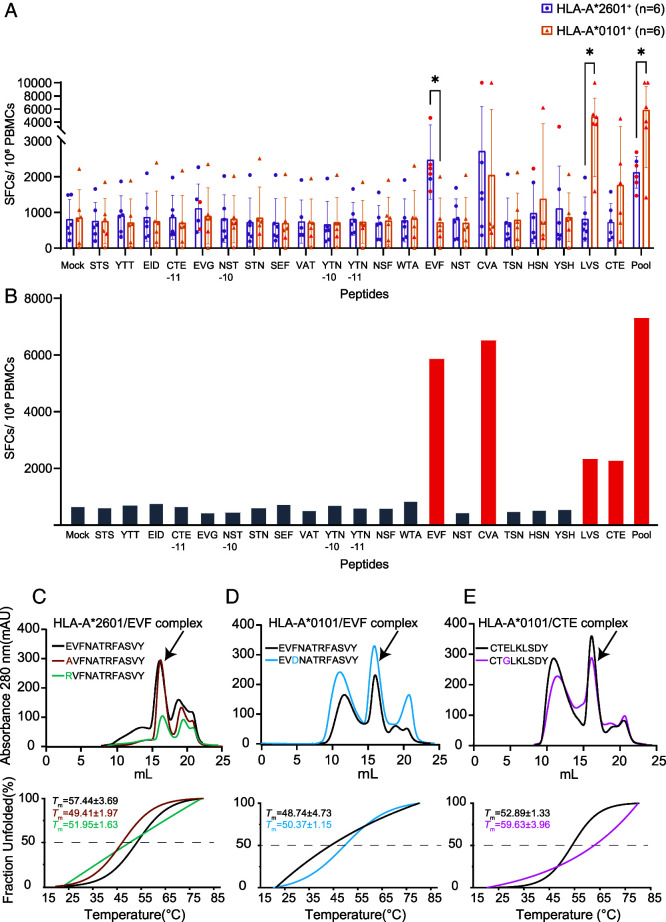
Different key residue preferences contribute to distinct T cell epitope recognitions among HLA-A*2601 and HLA-A*0101 donors. (**A**) Data analysis of the ELISPOT assay performed with HLA-A*0101 donors (*n* = 6, orange) and HLA-A*2601 donors (*n* = 6, purple). The error bars on each box in the chart represent the SE. ELISPOT dots twice larger than mock are shown as red dots. A Wilcoxon nonparametric test was used between groups, *p <* *0.05. (**B**) Data analysis of the ELISPOT assay performed with donor 34 (HLA-A*2601^+^ HLA-A*0101^+^). ELISPOT dots twice larger than mock are shown in the red column. (**C**–**E**) In vitro refolding (up panel) and CD (down panel) results of HLA-A*2601 binding to EVF (C), HLA-A*0101 binding to EVF (D), and HLA-A*0101 binding to CTE (E) and substitutions.

To further explore the similarities and differences between HLA-A*2601 and HLA-A*0101 presenting peptides, cross-conserved positive CD8^+^ T cell epitope peptides SARS-CoV-2 and IAV proteins were selected from the Immune Epitope Database and Tools (Table II). HLA-A*2601 and HLA-A*0101 donors were recruited with the HLA typing results (Table I). In the ELISPOT assays, four peptides, EVF, CVA, LVS, and CTE, showed immunodominance in the T cell responses among HLA-A*2601 and/or HLA-A*0101 donors. For the peptides from SARS-CoV-2, EVF is a 12mer peptide with negative-charged residue E in P1 position, and it stimulates higher IFN-γ production in HLA-A*2601. CVA stimulated both HLA-A*2601and HLA-A*0101, but there was no significant difference between two HLA allomorphs (Fig. 2A). For the peptides from influenza viruses, LVS and CTE with negative-charged residue E or D in P3/4 position stimulated higher T cell responses among HLA-A*0101 donors than HLA-A*2601, although no significance was observed for CTE. We also identified a donor with HLA-A*2601 and HLA-A*0101 heterozygosity and subsequently conducted the same ELISPOT assays on his PBMCs. Peptides testing positive for either HLA-A*2601 or HLA-A*0101 or both were observed (Fig. 2B).

To further determine the secondary anchor function of P1 or P3 of the peptides binding to HLA-A*2601 and HLA-A*0101, P1 or P3 of the peptide was mutated to Ala, which has short side chains, or Gly, which has no side chain, positively charged amino acid Arg, and/or negatively charged amino acid Asp. For EVF, when the original P1E mutated to oppositely charged R, it was observed that the peak intensity of in vitro refolding complexes of HLA-A*2601 decreased. When the P1E of EVF mutated to A, it led to a minor reduction (Fig. 2C). For HLA-A*0101, although EVF was not a positive peptide to induce T cell responses in donors tested, when the P3 position changes from F to D, the results of in vitro refolding indicate an increase in the quantity of the HLA-A*0101/EVF complex (Fig. 2D). CTE was an HLA-A*0101 positive peptide, and when the P3 position mutated from E to G, the quantity of HLA-A*0101/CTE complex decreased (Fig. 2E). The results of CD revealed typical stability unfolding degradation curves akin to those observed for HLA-A*0101/EVF-3-D, HLA-A*2601/EVF, and HLA-A*2601/EVF-1-A (Fig. 2C–2E, down panel), consistent with the in vitro refolding results. Fraction unfolding involves nonlinear fitting, and the original data are shown in Supplemental Fig. 1. These analyses indicate that the negative charges of the P1 anchor of HLA-A*2601-response epitopes and P3 of HLA-A*0101 epitopes are pivotal for the binding of the epitopes.

### Overall structures of HLA-A*2601 with pathogen peptides

To fully understand the specific features of peptide presentation by HLA-A*2601, we determined the four crystal structures of HLA-A*2601 complexed to SARS-CoV-2 T cell epitope peptides EVF and CVA and two peptides from HPV: DVM and YVA. We also determined one structure of HLA-A*0101 in comparison with HLA-A*2601 in detail (Table III).

**Table III.** X-ray data processing and refinement statistics

Parameter	HLA-A*2601/DVM	HLA-A*2601/YVA	HLA-A*2601/EVF	HLA-A*2601/CVA	HLA-A*0101/EVF-F3D
PDB code	8XES	8XFZ	8XG2	8XKC	8XKE
Data collection statistics					
Space group	C121	P212121	P212121	P212121	P212121
Cell parameters					
a(Å)	a = 167.12	a = 56.49	a = 60.27	a = 71.40	a = 53.17
b(Å)	b = 66.53	b = 70.83	b = 66.80	b = 80.87	b = 67.25
c(Å)	c = 49.33	c = 117.04	c = 116.82	c = 81.37	c = 122.47
α(˚)	α = 90.00	α = 90.00	α = 90.00	α = 90.00	α = 90.00
β(˚)	β = 90.23	β = 90.00	β = 90.00	β = 90.00	β = 90.00
γ(˚)	γ = 90.00	γ = 90.00	γ = 90.00	γ = 90.00	γ = 90.00
Resolution(Å)	27.58-1.78 (1.84-1.78)*^a^*	28.24-2.32 (2.40-2.32)	41.94-1.84 (1.94-1.84)	36.21-2.50 (2.29-2.18)	32.38-1.92 (1.99-1.92)
Total reflections	351,377	276,525	449,872	275,847	308,339
Unique reflections	52,088	21,060	48,349	25,558	25,142
Completeness (%)	99.5 (98.9)	100.0 (100.0)	100.0 (100.0)	100.0 (100.0)	99.6 (98.0)
R_merge_ (%)^b^	5.3 (48.5)	8.7 (95.0)	7.9 (68.6)	18.3 (460)	12.7 (173.6)
Ι/σ(I)	16.6 (2.5)	18.0 (2.6)	18.0 (3.1)	6.5 (1.2)	11.9 (1.5)
Refinement					
R_work_ (%)*^c^*	20.48	19.75	18.99	20.40	21.85
R_free_ (%)	23.49	25.07	22.96	28.20	25.52
r.m.s. deviation*^d^*					
Bonds(Å)	0.0066	0.008	0.0070	0.009	0.008
Angle(˚)	0.85	0.87	0.87	1.06	1.03
Average B factor(Å^2^)	34.0	53.0	31.0	67.0	41.0
Ramachandran plot quality					
Favored (%)	98.94	98.14	98.94	95.75	98.16
Allowed (%)	1.06	1.86	1.06	3.72	1.84
Outliers (%)	0	0	0	0.53	0

a Values in parentheses refer to statistics in the outermost resolution shell.

b R_merge_ = Σ_hkl_Σ_i_| I_i_ - | Σ_hkl_Σ_i_I_i_, where I_i_ is the observed intensity and is the average intensity of multiple observations of symmetry-related of reflections.

c R= Σ_hkl_ǁF_obs_ | -*k* | F_cal_ | |/Σ_hkl_ | F_obs_ |, where R_free_ is calculated for a randomly chosen 5% of reflections, and R_work_ is calculated for the remaining 95% of reflections used for structure refinement.

d r.m.s., root mean square.

**FIGURE 3.**
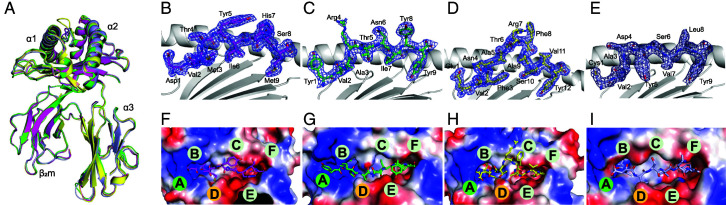
Overview structure of the HLA-A*2601 complex. (**A**) Overall structure of HLA-A*2601/peptide complex. (**B**–**E**) Electron densities and overall conformations of the DVM (B), YVA (C), EVF (D), and CVA (E) complexed with HLA-A*2601. Simulated annealing omits maps (CNS) calculated for the four peptides are shown in blue at a contour of 1.0σ viewed in profile through the α2 helix. The electron density map was constructed from model phases, omitting the peptide. The peptides are displayed as sticks. (**F–I**) Vacuum electrostatics surface potential of PBG of DVM (F), YVA (G), EVF (H), and CVA (I) complexed with HLA-A*2601. The blue surface represents the positive potential, and the red surface represents the negative potential; the six pockets on the PBG are marked with the letters A to F.

The overall structure of HLA-A*2601 displays the common characteristics of classic HLA-I molecules, with the extracellular region of the H chain folding into three domains. The α1 and α2 domains construct a typical PBG that contains two α-helices and eight β-sheets, and the α3 domain and β_2_m display typical Ig domains and underpin the PBG (31) (Fig. 3A). The all-atom superimposition of four structures showed similar overall conformations, with root mean square deviation of 0.374 Å, 0.389 Å, and 0.392 Å to HLA-A*2601/EVF, which indicated that these molecules were extremely similar. The electron density maps contoured at 1.0σ showed the solid convincing conformation of the peptides binding to HLA-A*2601 (Fig. 3B–3E). The Fo-Fc omitted maps of peptides are shown in Supplemental Fig. 2. The vacuum electrostatic surface clearly exhibited the six pockets in the PBG of HLA-A*2601 to accommodate the peptides. P2 residues of the peptides insert into B pockets, and the side chains of P9 protrude into F pockets. Notably, obvious positively charged P1 can be observed in half of the structures of HLA-A*2601 to accommodate the P1 anchors of the peptides (Fig. 3F–3I).

### Shallow pocket C of HLA-A*2601 leads to variable conformations of peptides

**FIGURE 4.**
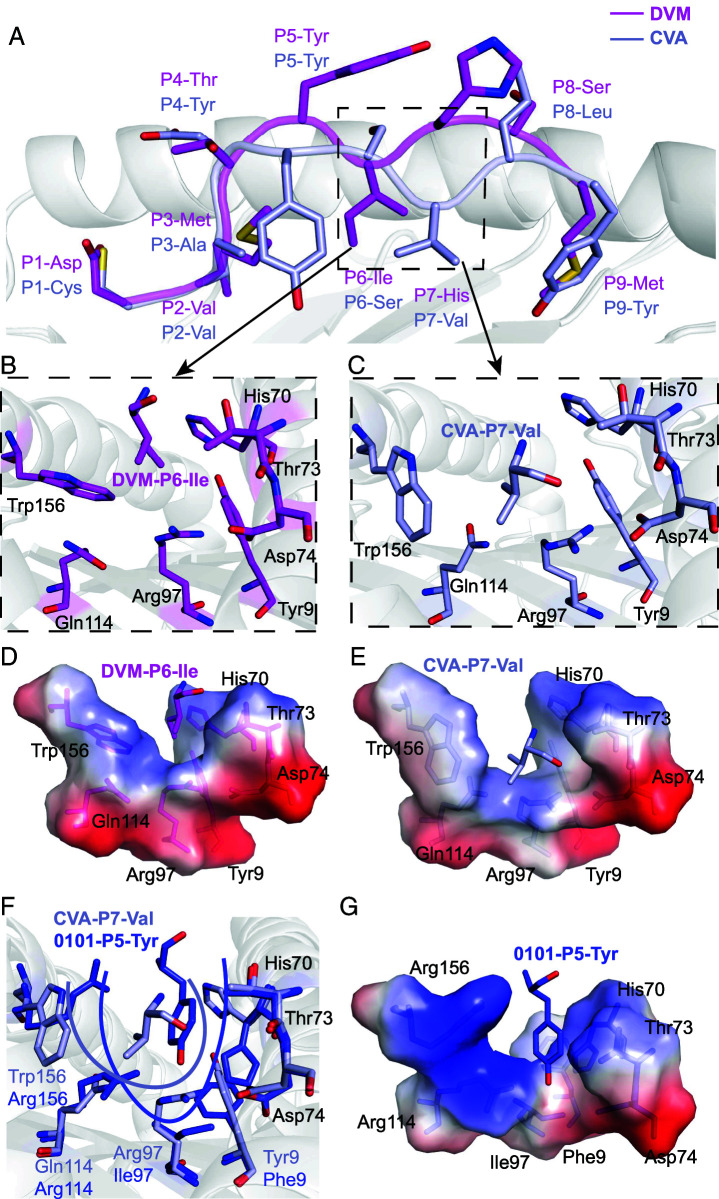
Switchable bulged conformation of HLA-A*2601 presenting peptides. (**A**) Overall structure of peptide binding HLA-A*2601. (**B** and **C**) Insertion of the sixth amino acid (**B**) or the seventh amino acid (**C**) into the pocket C results in a “switchable” conformation of the peptide. The major residues are shown as sticks. (**D** and **E**) The vacuum electrostatic surface shows the key residue that determine the size of the pocket C. (**F**) Comparison of key residues in pocket C between HLA-A*2601/CVA (light blue) and HLA-A*0101(PDB code 6AT9, blue). The names of similar residues are labeled in black; the different ones are labeled according to the colors of the HLA-A allomorphs. (**G**) Surface of pocket C of HLA-A*0101.

The four peptides presented by HLA-A*2601 showed diverse conformations, although P2 and P_Ω_ act as main anchor residues for all the peptides (Fig. 3B–3E). As a long 12-mer peptide, EVF has a superbulged conformation with the residues Thr6, Arg7, Phe8, and Ala9 exposed as a long loop outside the PBG of HLA-A*2601. For the other three peptides, although CVA, DVM, and YVA are all nonapeptides, they show different conformations with residues in P6 or P7 of peptides as the secondary anchors (Fig. 4A). In the structure of HLA-A*2601/CVA, the P7-Val side chain of the peptide points toward the pocket C, whereas side chains of P6-Ser and P8-Leu orient upward, away from the PBG. Meanwhile, for DVM, the side chain of P6-Ile inserts into the pocket C, and the side chains of P5-Tyr and P7-His arch upward, forming the “switchable” bulged conformation due to different position of secondary anchor residue inserted (Fig. 4A). This is due to the fact that the pocket C is shallow due to Tyr9, His70, Thr73, Asp74, Arg97, and Gln114 with long and large side chains in the PBG, and Trp156 contributes to the volume of pocket C. For CVA, the side chain of P7-Val can insert into the pocket C (Fig. 4C, 4E). But for DVM, the large side chains in P5-Thr and P7-His result in potential significant steric obstruction if they insert into pocket C. Instead, P6-Ile with smaller hydrophobic side chain points into the pocket C (Fig. 4B, 4D). Actually, peptide YVA in HLA-A*2601 also performs a similar conformation with CVA, while P7-Ile as the secondary anchor (Supplemental Fig. 3).

Four amino acid changes between HLA-A*2601 and HLA-A*0101 resulted in the distinctive shallowness of pocket C in HLA-A*2601. The bottom of pocket C is determined by Arg97. Compared with HLA-A*0101, the side chain of Ile97 in HLA-A*0101 is small, creating a deep pocket. In contrast, the side chain of Arg97 in HLA-A*2601 orients upward, thereby elevating the bottom of pocket C and reducing its volume. Simultaneously, the imidazole moiety of His70 in HLA-A*0101 rotates downward, forming the right side of pocket C. In contrast, the imidazole moiety of His70 in HLA-A*2601 points toward the pocket, although Trp156 is in α2 helix and is the component of pocket D, but its indole moiety is inserted into the pocket, forming the left side of the pocket C, together further contributing to the reduction in pocket C volume (Fig. 4F–4G). For quantitative and precise description of the variation in the size volume of pocket C, we used https://proteins.plus/ to calculate the depth and volume of the pocket. The depth of pocket C in 6AT9 is 20.41 Å, and the volume is 701.95 Å^3^, and for pocket C in HLA*2601/DVM, the depth is 17.64 Å, and the volume is 635.90 Å^3^.

### Distinct peptide motifs depended on differently charged pockets of HLA-A*2601 and HLA-A*0101

**FIGURE 5.**
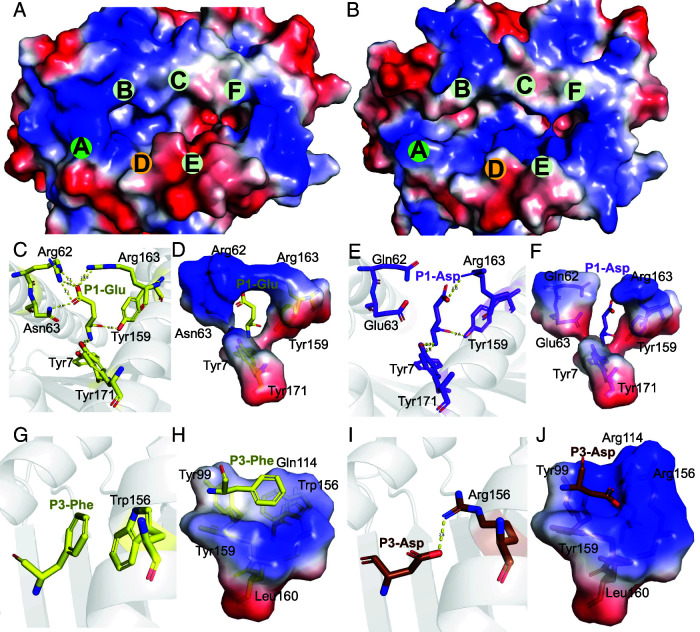
Pockets A and D comparison between HLA-A*2601 and HLA-A*0101. (**A**) The vacuum electrostatic surface of HLA-A*2601/EVF. (**B**) The vacuum electrostatic surface of HLA-A*0101 (PDB code 1W72). Only the surface of H chain is displayed. (**C**, **D**) Key residues in pocket A in HLA-A*2601/DVM. (**E** and **F**) Key residues in pocket A in HLA-A*0101 (PDB code 1W72). (**G** and **H**) Key residues in pocket D in HLA-A*2601/EVF. (**I** and **J**) Key residues in pocket D in HLA-A*0101/EVF-F3D. Hydrogen bonds are shown as yellow dashed lines.

HLA-A*2601 and HLA-A*0101 prefer negatively charged secondary anchor residues for the P1 and P3 positions of the peptides, respectively, which may be determined by the amino acids differing in α1α2 domains between HLA-A*2601 and HLA-A*0101 (Fig. 1A). Pockets A and D exhibit electrostatic preference differences between HLA-A*2601 and HLA-A*0101 (Fig. 5A, 5B). Pocket A of HLA-A*2601 obviously presents a positive-charged surface. At position 62, the unique residue Arg in HLA-A*2601, along with Arg163, forms a salt bridge network with P1, thereby stabilizing P1 of the peptide. Meanwhile, three Tyr residues, Tyr7, Tyr159, and Tyr171, establish hydrogen bonds with the free −NH_3_ on the main chain of the P1 amino acid, contributing to the anchoring of P1 in the pocket A (Fig. 5C, 5D). Meanwhile, the residue Gln62 of HLA-A*0101 cannot constitute the positive-charged pocket A, although Tyr7, Tyr159, and Tyr171 can still contribute to the anchoring of N terminus of the peptide (Fig. 5E, 5F).

The pocket D of HLA-A*2601, influenced by the Trp156, lacks a preference for peptide-binding charge (Fig. 5G, 5H). In contrast, for HLA-A*0101, Arg156 can form a salt bridge with P3-Asp or Glu of the peptides, and the Arg114 further enhances the positive charge of the pocket D (Fig. 5I, 5J). Notably, the third position of the peptide in HLA-A*0101 structures [PDB codes 1W72 (32), 4NQV (28), 6AT9 (33) and 6MPP] available in the PDB consistently features D or E (Supplemental Fig. 4).

**FIGURE 6.**
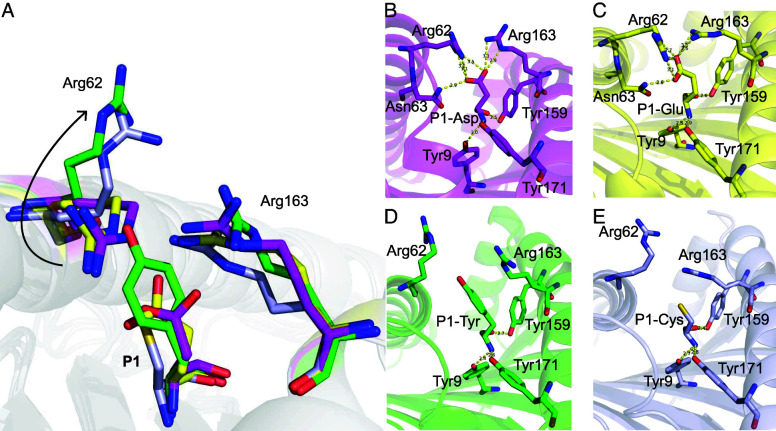
Closed and open conformation for the P1 anchors within the HLA-A*2601 pocket A. (**A**) The conformational flip of Arg62. (**B** and **C**) Closed conformation of DVM (magenta) and EVF (yellow) in HLA-A*2601 complex. (**D**) Open conformation of YVA (green) and CVA (light blue) in HLA-A*2601 complex. Key residues are shown in stick. Hydrogen bonds are shown as yellow dashed lines.

Due to the diversity of peptides that can be bound in the PBG, residues on HLA may have a variety of conformations. The results from random peptides indicate that the P1 position may not be exclusively occupied by D or E, suggesting potential variability in the conformation of pocket A. (Fig. 6A). When P1 is D or E, as described above, it leads to the pulling of Arg62 and Arg163 toward P1, resulting in the “close” conformation of pocket A. At this point, P1 is firmly anchored within pocket A (Fig. 6B, 6C). As in HLA-A*2601/YVA with P1-Tyr and HLA-A*2601/CVA with P1-Cys, due to the absence of negative charges, Arg62 and Arg163 of HLA-A*2601 are not drawn toward the P1 site. As a result of repulsion between Arg62 and Arg163, Arg62 rotates upward, moving away from the PBG, causing the “open” conformation of pocket A (Fig. 6D, 6E). This explains the preference of HLA-A*2601 for negatively charged amino acids at P1 while also explaining that pocket A produces different conformations for different P1 amino acids.

### Conserved pocket F binding preference between HLA-A*2601 and HLA-A*0101

**FIGURE 7.**
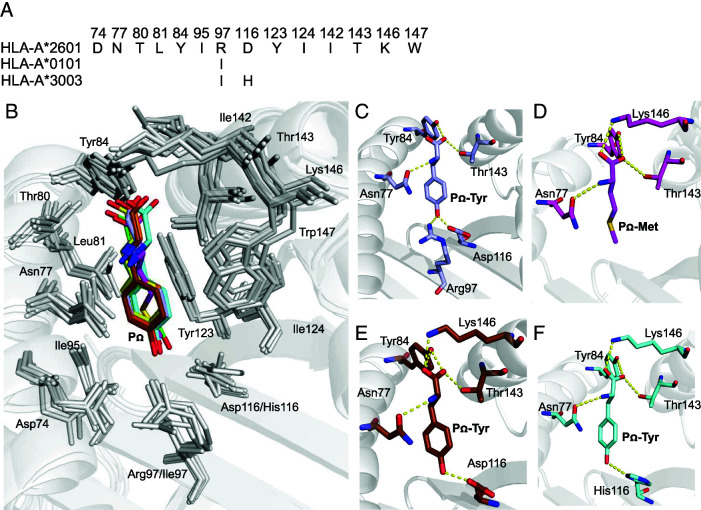
Conserved pocket F binding preference between HLA-A*2601 and HLA-A*0101. (**A**) Alignment of key residues composing the F pocket of allomorphs possessing the same characteristic binding motif. HLA-A*3003 GenBank no. ANG08799.1. (**B**) Key residues in pocket F. P_Ω_ of HLA-A*2601 complex with DVM (magenta), YVA (green), EVF (yellow), and CVA (light blue), HLA-A*0101 complex with EVF-E3D (brown), HLA-A*3003 complex (PDB code 6J2A, cyan) are shown in stick. (**C**) Key residue of pocket F in HLA-A*2601 complex with CVA. (**D**) Key residue of pocket F in HLA-A*2601 complex with DVM. (**E**) Key residue of pocket F in HLA-A*0101 complex with EVF-E3D. (**F**) Key residue of pocket F in HLA-A*3003 complex (PDB code 6J2A).

The P_Ω_ position of the HLA-A*2601-binding peptides exhibits a preference for large hydrophobic amino acids, similar to the binding motif of HLA-A*0101, as well as A1A3 superfamily (34) molecules, such as HLA-A*3003. The residues comprising pocket F exhibit significant conservation among HLA-A*2601, HLA-A*0101, and HLA-A*3003 (Fig. 7A). In the structures of HLA-A*2601 complexed to EVF, CVA, and YVA, the P_Ω_ position is occupied by Tyr, forming hydrogen bonds with residues Asn77, Tyr84, and Lys146 in the HLA. Asp116 forms a hydrogen bond with the phenolic group of the side chain of P_Ω_-Tyr, thereby stabilizing the P_Ω_ anchoring and conformation (Fig. 7C, Supplemental Fig. 5). pocket F, characterized by a substantial volume, favors the accommodation of large side-chain amino acids such as Tyr (Fig. 7E, 7F). Interestingly, in the HLA-A*2601/DVM structure, the P_Ω_ position is occupied by Met. The P_Ω_-Met inserts into pocket F and forms similar hydrogen bonds with the peptide backbone of Asn77, Tyr84, and Lys146, thereby stabilizing the P_Ω_ conformation (Fig. 7D). This observation highlights the versatility of the F pocket in accommodating both tyrosine and methionine residues.

## Discussion

**FIGURE 8.**
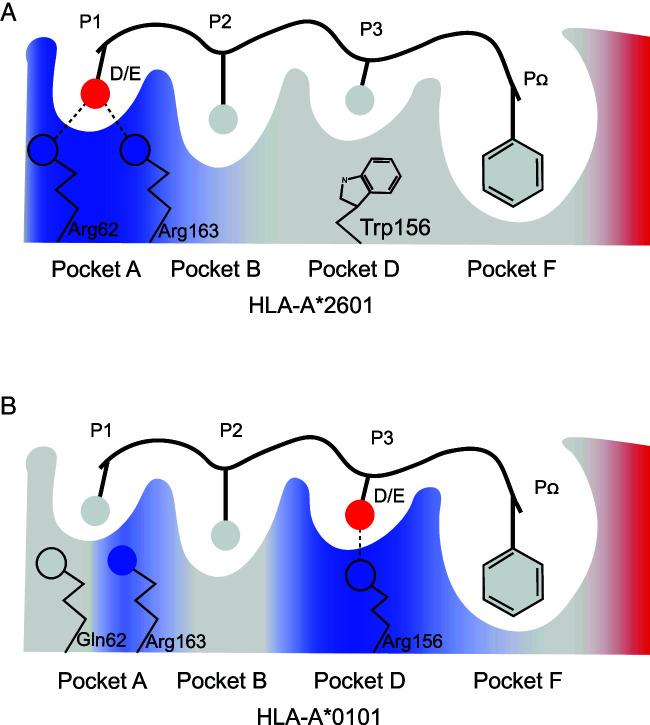
Peptide presenting model by HLA-A*2601 and HLA-A*0101. Peptide binding model for HLA-A*2601 (**A**) and HLA-A*0101 (**B**). The critical A, B, D, and F pockets are shown, Main chain of peptides is shown in line, and only the side chains are shown. Blue represents the positive potential, red represents the negative potential, and gray represents the polarity potential. Hydrogen bonds are shown as black dashed lines.

In this study, we present the uncommon presentation and T cell recognition on the T cell epitopes with acidic amino acids of among the HLA-A*2601 individuals. HLA-A*2601 and HLA-A*0101 showed distinct peptide motif preference and T cell responses, which are elucidated through a series of structural and functional investigations (Fig. 8). Furthermore, HLA-A*2601 can accommodate variable peptide conformations due to the shallow and narrow pocket C because of its minor polymorphisms. These data present the special T cell epitope presentation and recognition among the prevalent population with the HLA-A*2601 allomorph and may shed light on the T cell–based immune intervention.

Over time, the division of HLA allomorphs into specific superfamilies has been based on a variety of methods. We reviewed and found there are numerous reports on the superfamily classification of HLA-A*2601. Sette et al. first classified HLA-A*2601 into the A1 superfamily based on the P2 and P_Ω_ and defined the A1-supermotif as (TSIVLM)_2_ and (YWF)_c_ (7). Sidney et al. also classified HLA-A*2601 into the A1 superfamily based on the peptide preferences for the B and F pockets of A1 (8). Later, Doytchinova et al. put HLA-A*2601 into the A2 or A3 superfamilies (35). Naugler et al. found the HLA-A1 superfamily has five independent origin clades from HLA-A3 superfamily ancestors (36). Nevertheless, Shen et al. suggested that HLA-A*2601 belongs to the A66 subtype and HLA-A*0101 belongs to A01 subtype; both alleles belong to the A1-A3-A66 superfamily (37). Our study provides, to some extent, experiment-based insights into the classification of HLA-A*2601 and HLA-A*0101.

Our findings indicate that the positively charged pocket A of HLA-A*2601 prefers the accommodation of peptides with acidic amino acids in the P1 position (Fig. 5). As indicated in the previous studies, negative amino acids at the P1 position of peptide occurred during normal proteasomal processing in cells, and the acidic amino acids D and E can be enriched at P1 in peptides generated by constitutive proteasomes (38). Therefore, the preferred presentation of peptides with acidic amino acids in the P1 position of HLA-A*2601 may play a pivotal role in antiviral immunity by virtue of the high abundance of viral peptides with acidic amino acids at the N-terminus. Our results also explained the molecular basis for the solid anchoring of the negatively charged P1 residues of the HLA-A*2601 peptides. As one the allomorph-specific residues, Arg62 can form a tight hydrogen bond network with the either D or E of P1 of the binding peptides in the assistance of Arg163. In contrast, the P3 position of HLA-A*0101 binding peptide exhibits a strong bias for negatively charged amino acids (Fig. 5) (39), and two acidic acid residues at P1 act as secondary anchors for HLA-A*2601 (40).

Actually, although P1 residues of the T cell epitopes do not act as a major anchor for the peptide presentation by MHC I, a series of structures of MHC I from diverse species indicate that the P1 anchor can play an important role in the MHC I/peptide binding (41, 42). However, it shows a diverse binding mode for the interaction between the P1 anchors and the amino acids within pocket A. In our previous study of the first bat MHC I molecule structure Ptal-N*0101, the interaction between P1-Asp and Arg65, along with Asp59, resulted in the formation of a triangular hydrogen bond network. This can be attributed to the unique feature that three amino acids insert in α1-helix in bat MHC I. Consequently, the bat-specific Asp59 protrudes into pocket A, forming the hydrogen bond network that securely anchors P1-Asp within pocket A. This suggests that P1-Asp can act as a “surface anchor residue” (41). Although both Ptal-N*0101 and HLA-A*2601 exhibit a preference for acidic amino acids at the P1 position, their molecular mechanisms differ. Furthermore, crystal structure and in vitro refolding of the feline MHC I FLA-E*01801 demonstrated that Glu63 and Trp167 in pocket A play important roles in the preference of P1-Lys/Arg and the restriction of P1-Asp (42). This indicates the importance of charged amino acid pairing for interactions, although the P1 anchor and the residues in pocket A of FLA-E*01801 have an opposite electrification compared with our currently determined structure of HLA-A*2601.

The structures of HLA-A*2601 complexed to the three 9-mer peptides (i.e., DVM, YVA from HPV, and CVA from SARS-CoV-2) revealed diverse conformations of the binding peptide in the median portion. Only the residues with short side chain in the middle portion of the peptides (such as P6-Ile of DVM and P7-Val of CVA) can be accommodated in the shallow pocket C. This led to diverse conformations for the HLA-A*2601 if the residues with short side chain are located in the different positions of peptides. In the structure of the 12-mer EVF peptide presented by HLA-A*2601, with P2 and P_Ω_ serving as anchor residues, whereas P5 to P9 protrude from the HLA, forming a loop available for TCR recognition. Moreover, this peptide exhibits a positivity rate of 85.7% (6 of 7; Fig. 2A, 2B) within the HLA-A*2601 population. Tynan et al. analyzed the structure of 13-mer peptide presented by HLA-B*3508 and revealed that the CDR3β region crosses over the tip of the bulged epitope, and P7-Gln extends deeply into a pocket within the TCR, forming Ab-like interactions (43). The importance of HLA-I presentation of bulged peptides has been emphasized (44).

Meanwhile, our results provide not only insights into the conformational flexibility of the complex but also a better understanding of the molecular immunological characteristics due to the minor polymorphism in HLA H chain. MHC I/peptide complex recognized by TCR can initiate T cell immune signal transduction. Anand et al. found that TCR recognition of HLA-C*0602 presenting peptides was mainly concentrated on three amino acids of the peptide, P4-Arg, P5-Arg, and P7-Arg, with upward side chain orientation (45). Chaurasia et al. showed that CDR3β Asn110 of the TCR forms hydrogen bonds with the peptide as well as the HLA-A2 α2 domain. However, this HLA I–restricted TCR repertoire is highly sensitive to peptide variants (46). Changes in the conformation of the peptide presented by HLA-A*2601 may lead to changes in the TCR recognition, which needs further investigation.

For CTE, if mutated, the third position mutated to G in H1Nx (Supplemental Table I). Our results showed that refolding decreased after P3G mutation. Mutations of this peptide at the third position in the natural environment may result in decreased T cell responses to it in HLA-A*0101 people, leading to immune escape from IAV. Interestingly, CTE can also be presented by swine SLA-1*0401 (47). IAV as a zoonotic virus, a peptide that is recognized by both human and animal MHC, could provide valuable knowledge in terms of future IAV vaccine design. For CVA, it can be presented by not only HLA-A*0101 and HLA-A*2601 but also HLA-A*2902 (48) and HLA-B*1501 (49). The epitope can be cross-presented by multiple HLA allomorphs (50), which can serve as targets for the design of universal vaccines.

Through the comparative analysis between HLA-A*2601 and HLA-A*0101, we elucidate the distinct features of the binding peptides and the T cell responses among these two allomorphs. Our data are helpful for the understanding of CD8^+^ T cell recognition among different HLA populations and may provide new insight into T cell–oriented vaccine development.

## Supplementary Material

Supplemental Material (PDF)
