# Supplementary material for: Uncommon P1 Anchor-featured Viral T Cell Epitope Preference within HLA-A*2601 and HLA-A*0101 Individuals
Source: Immunohorizons. 2024 Jun 17;8(6):415–30. doi: 10.4049/immunohorizons.2400026 (PMC11220742; doi:10.4049/immunohorizons.2400026)
Supplement: Supplemental Material (PDF) [file IH_2400026_Supplemental_1.pdf]

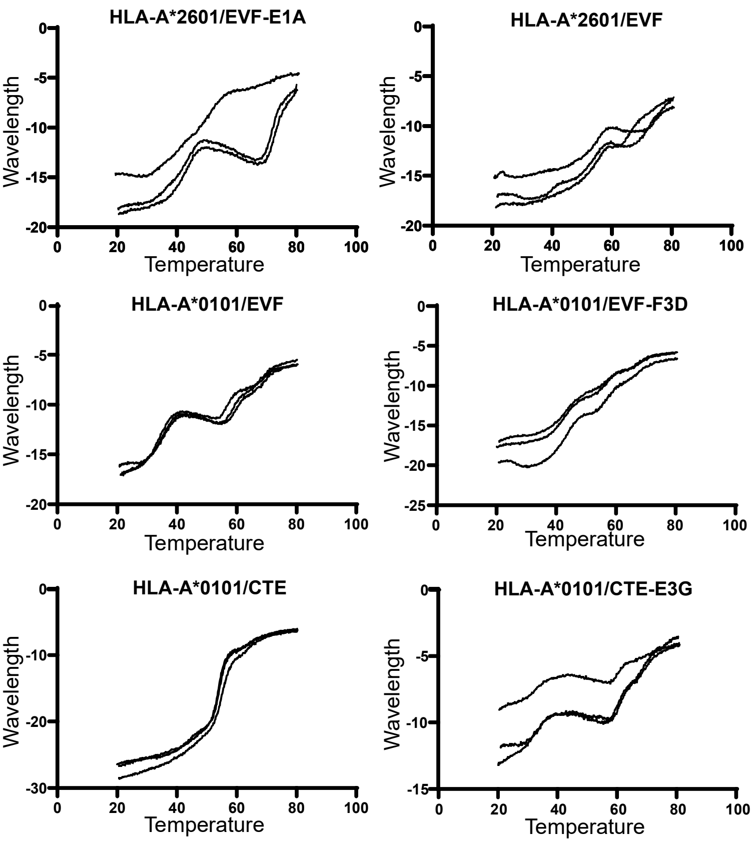

Figure S1. The CD original data of HLA/peptide complexes.
CD spectra of HLA-A*0101 and HLA*2601 complexed with peptides are collected at the wavelength of 218nm and recorded in 20mM Tris, 50mM NaCl buffer(pH=8).

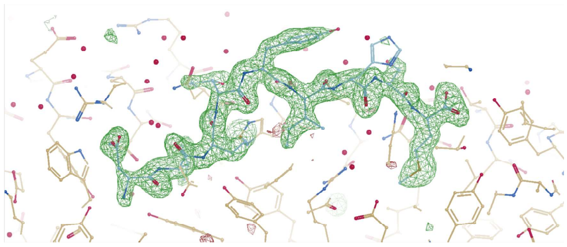

HLA-A*2601/DVM

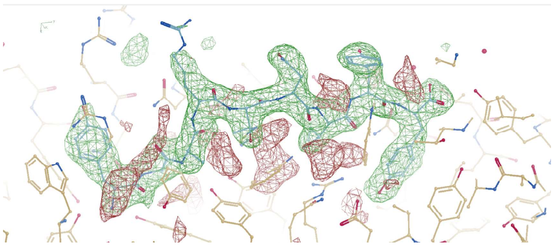

HLA-A*2601/YVA

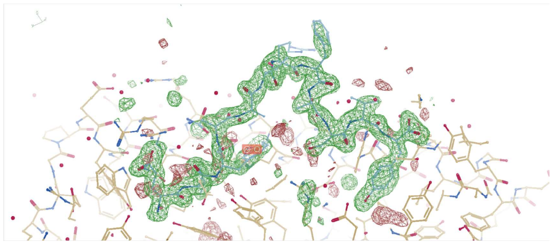

HLA-A*2601/EVF

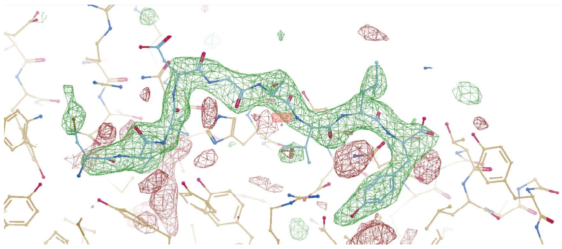

HLA-A*2601/CVA

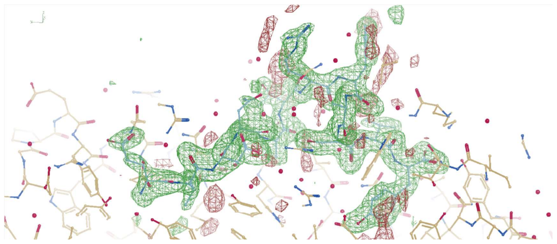

HLA-A*0101/EVF-F3D

Figure S2. Omit Map Analysis of peptides in HLA/peptides complexes.
The omit map analysis performed for five HLA-A/peptide complex. Greem mesh represents electron density at contour level 3σ, overlaid on the molecular structure. The chain of peptide showed in green.

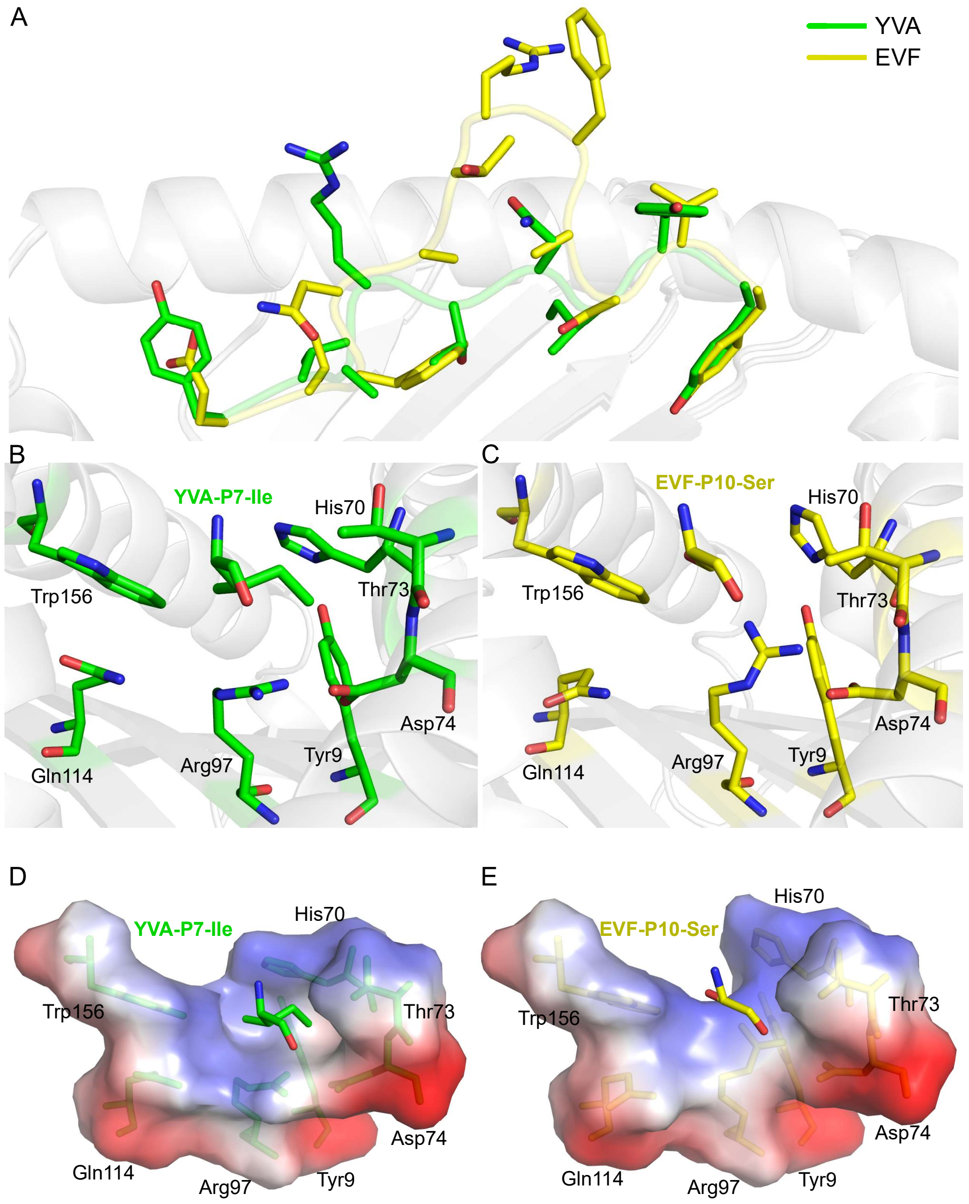

Figure S3. Pocket C of HLA-A*2601/YVA and HLA-A*2601/EVF complexes. **A**: Overall structure of peptide binding HLA-A*2601. **B-C**: The different bulged conformation of HLA-A*2601/YVA(**B**) and HLA-A*2601/EVF(**C**). The major residues are shown as sticks. **D-E**: The vacuum electrostatic surface shows the key residue that determine the size of the pocket C.

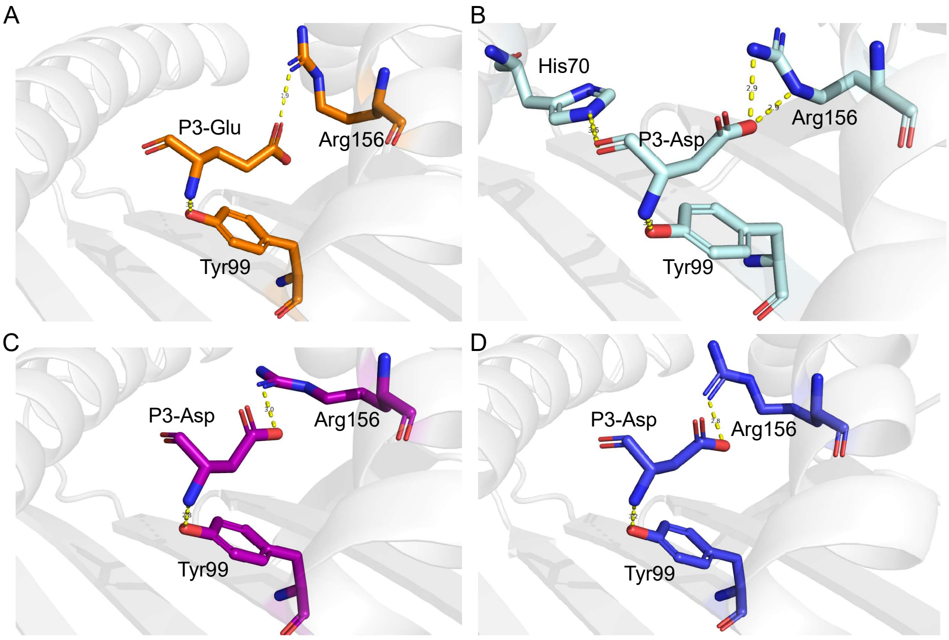

Figure S4. Electrostatic character of P3 in HLA-A*0101 binding peptides. HLA-A*0101/peptide structures PDB code: 4NQV (**A**, orange), 6MPP (**B**, pale cyan), 1W72 (**C**, purple), 6AT9 (**D**, blue). P3 of peptides and interacting amino acid residues in HLA-A*0101 are shown in sticks. Hydrogen bonds are shown as yellow dashed lines.

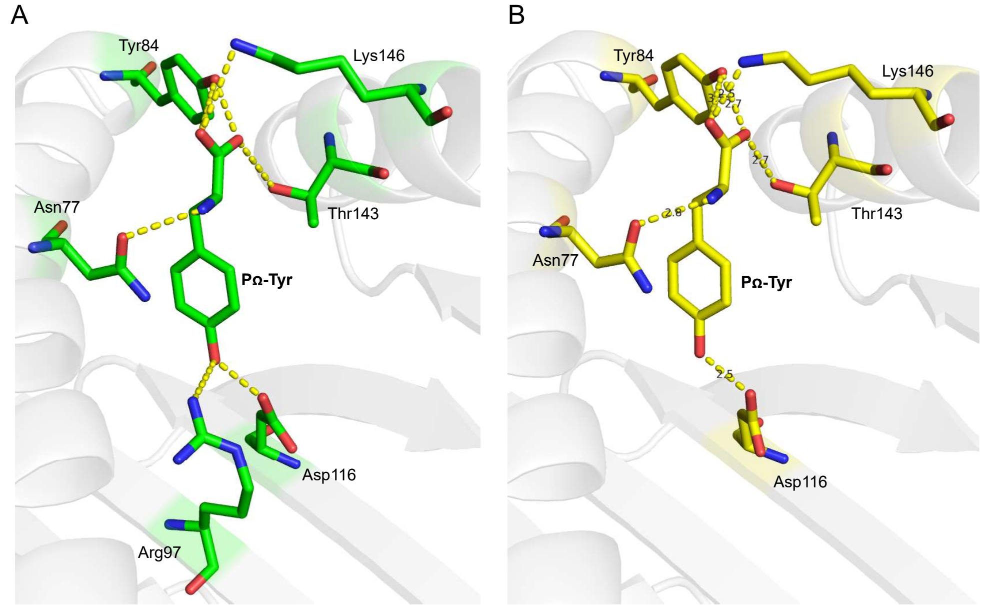

Figure S5. Pocket F of HLA-A*2601/YVA and HLA-A*2601/EVF complexes. **A**: Key residue of pocket F in HLA-A*2601/YVA. **B**: Key residue of pocket F in HLA-A*2601/EVF. PΩ of peptides and interacting amino acid residues are shown in sticks. Hydrogen bonds are shown as yellow dashed lines.

Supplemental Table I. Mutation of CTE (IAV NP44-52) under natural conditions

Position	Reference[a](frequency)	Mutation(frequency)
1	C (99.99532%)	R (100%)
2	T (100%)	
3	E (99.99352%)	G (100%)
4	L (99.98056%)	I (66.67%), F (33.33%)
5	K (95.44512%)	Q (97.87%), R (2.13%)
6	L (99.95352%)	F (100%)
7	S (89.62032%)	N (98.81%), G (0.56%), D (0.31%), R (0.12%), I (0.06%), T (0.06%), X (0.06%)
8	D (99.95465%)	E (71.43%), N (28.57%)
9	Y (99.45575%)	H (92.86%), N (3.57%), C (2.38%), Q (1.19%)

[a] Refers to the amino acid with the highest frequency at NP44-52 of the Influenza A virus H1Nx sequence, among a total of 15,435 entries retrieved from the NCBI (https://www.ncbi.nlm.nih.gov/).
